# Neuropeptide Y and Peptide YY in Association with Depressive Symptoms and Eating Behaviours in Adolescents across the Weight Spectrum: From Anorexia Nervosa to Obesity

**DOI:** 10.3390/nu13020598

**Published:** 2021-02-11

**Authors:** Marta Tyszkiewicz-Nwafor, Katarzyna Jowik, Agata Dutkiewicz, Agata Krasinska, Natalia Pytlinska, Monika Dmitrzak-Weglarz, Marta Suminska, Agata Pruciak, Bogda Skowronska, Agnieszka Slopien

**Affiliations:** 1 Department of Child and Adolescent Psychiatry, Poznan University of Medical Sciences, 61-701 Poznan, Poland; katarzyna.jowikk@gmail.com (K.J.); dutkiewicz.agata22@gmail.com (A.D.); adnat@wp.pl (N.P.); asrs@wp.pl (A.S.); 2 Department of Pediatric Diabetes and Obesity, Poznan University of Medical Sciences, 61-701 Poznan, Poland; agatakrasinska@gmail.com (A.K.); marta_suminska@o2.pl (M.S.); bskowron@ump.edu.pl (B.S.); 3 Psychiatric Genetics Unit, Department of Psychiatry, Poznan University of Medical Sciences, 61-701 Poznan, Poland; m.weglarz1@gmail.com; 4 Institute of Plant Protection—National Research Institute, Research Centre of Quarantine, Invasive and Genetically Modified Organisms, 60-318 Poznan, Poland; apruciak93@gmail.com

**Keywords:** anorexia nervosa, obesity, neuropeptide Y family

## Abstract

Neuropeptide Y (NPY) and peptide YY (PYY) are involved in metabolic regulation. The purpose of the study was to assess the serum levels of NPY and PYY in adolescents with anorexia nervosa (AN) or obesity (OB), as well as in a healthy control group (CG). The effects of potential confounders on their concentrations were also analysed. Eighty-nine adolescents were included in this study (AN = 30, OB = 30, and CG = 29). Anthropometric measurements and psychometric assessment of depressive symptoms, eating behaviours, body attitudes, and fasting serum levels of NPY and PYY were analysed. The AN group presented severe depressive symptoms, while the OB group held different attitudes towards the body. The levels of NPY were lower in the AN and OB groups as compared with the CG. The PYY levels were higher in the OB group than in the AN group and the CG. The severity of eating disorder symptoms predicted fasting serum concentrations of NPY. Lower levels of NPY in AN, as well as in OB suggests the need to look for a common link in the mechanism of this effect. Higher level of PYY in OB may be important in explaining complex etiopathogenesis of the disease. The psychopathological symptoms may have an influence on the neurohormones regulating metabolism.

## 1. Introduction

Eating disorders (EDs) and obesity (OB) are distinct conditions with several overlapping biopsychosocial risk factors, such as ethnicity, personality traits, adverse events, and neurobehavioural disturbances [1,2]. Globally, the prevalence of overweight in the paediatric population is increasing rapidly. It is estimated that 170 million children have a weight exceeding the norm. The childhood OB epidemic is a serious problem, not only regarding physical health, but also the development of mental disorders such as depression or EDs [3]. Recent studies have highlighted OB and anorexia associated with medical conditions, as well as OB and EDs as potentially being two sides of the same coin [4,5,6]. The brain structures and functions of patients with OB and metabolic disorders contrast to those seen in patients with anorexia nervosa (AN) [7]. Studies have identified significant negative genetic correlations between AN and OB, suggesting that these conditions may represent “metabolic bookends” [8]. Moreover, a recent genome-wide association study (GWAS) resulted in the reconceptualisation of AN, i.e., it is not just a mental illness, but a metabo-psychiatric disease [9], which allows us to compare it with other metabolic disorders, including OB. AN is characterised by a disturbed body image, an extreme fear of weight gain, and weight loss, mostly due to restricted food intake. It has a chronic course and a high mortality rate. It also reduces the patient’s quality of life and places an emotional burden on individuals and families.

The aetiology of both AN and OB is still poorly understood, and treatment programmes leave a lot to be desired. In recent years, several peptides and hormones crucial for maintaining homeostasis through the regulation of appetite and metabolism have been identified. Many of them are produced and interplay in the brain, fat tissue, or digestive system. Moreover, some of them act in the limbic system and cerebral cortex, regulating perception, mood, motivation, and other food-related processes [10,11,12]. The Y neuropeptide family seems particularly relevant because it has a significant influence on the regulation of food and metabolism, both at the central and peripheral levels, and is involved in gut–brain communication. It includes neuropeptide Y (NPY), peptide YY (PYY), and pancreatic polypeptide (PP).

PYY is secreted mainly in the gastrointestinal system, by L-type neuroendocrine cells located in its lower part and by the endocrine cells of the pancreas [13,14,15,16]. It is produced in proportion to the number of calories consumed; however, the type of food is also crucial for its secretion. PYY concentration increases shortly after a meal and stays elevated for several hours [17,18]. PYY plays a physiological role in the central and peripheral regulation of appetite, signalling the end of a meal, reducing food intake, and slowing gastrointestinal tract motility [19]. A single animal study suggested that PYY receptors are distributed within the hypothalamic arcuate nucleus, preoptic and dorsomedial nuclei, nucleus tractus solitarii of the brain stem [20], medial nucleus of the amygdala, substantia nigra, and parabrachial area [21]. Thus, PYY action on the limbic system (including the amygdala) might be significant when considering the modulation of hedonic pathways [20].

In contrast, NPY is a peptide produced and acting mainly in the brain. NPY and its receptors are widely expressed in the cerebral cortex, thalamus, hippocampus, amygdala, and basal ganglia [22,23,24]. However, they are also secreted in the enteroendocrine cells of the intestine, sympathetic neurons of blood vessels, lymph tissue, and immune cells. NPY is one of the most potent orexigenic peptides in the brain. It reduces energy expenditure and causes weight gain after intracerebral, but not peripheral, administration [25,26,27]. It is known that leptin is a potent inhibitor of NPY secretion [28]. Moreover, animal studies have suggested that NPY plays a significant role in the entero-cerebral axis. For instance, NPY expression was shown to be higher in the hypothalamus of germ-free mice than in animals fed traditionally [29]. NPY is also involved in regulating mucosal permeability, blood circulation, and intestinal peristaltic movements [30,31,32]. Peripherally, it promotes adipogenesis and inhibits lipolysis.

It has been postulated that both neuropeptides NPY and PYY interplay in homeostasis regulation. Thus, in NPY/PYY-knockout mice, lower food intake and impaired insulin action have been observed [30,31,32]. Furthermore, in recent years, researchers have emphasised the role of NPY in the regulation of anxiety, cognition, mood, and stress levels [33,34,35]. In rats, amygdala-based NPY administration has shown a reduction in anxiety and depressive behaviours [36,37], while NPY-knockout mice have been shown to present anxiety and depressive behaviours [38,39]. Similarly, a depression-like phenotype has been observed in PYY-knockout mice [39]. Animals under the influence of a stressor show a negative correlation of PYY levels with motivation and apathy-like behaviours [40]. Moreover, the neuroprotective, neuroproliferative, and neurogenesis-induced effects of NPY have been proven [41]. Peptides from the NPY family are unique. NPY is one of the most influential agents in stimulating food intake, and PYY is released in response to calorie intake, thereby inducing satiety. Moreover, both might be important for the functioning of the brain–gut axis and might also participate in non-homeostatic food intake regulation.

Human studies on NPY and PYY in OB and AN are inconclusive. Most of them have focused on the role of NPY in overweight subjects. They have proven that the NPY gene is associated with increased body weight and that NPY activity is increased in OB [42]. Less is known about PYY; some studies have shown its concentration to be reduced, but others have not confirmed this [43]. Moreover, only a few studies have been conducted among patients with AN, usually including a small number of adult patients. In these studies, both PYY and NPY were discovered to be unchanged, increased, or decreased, as discussed later in this article.

There is a gap in the knowledge of hormonal appetite responses in adolescents with either OB and AN. Studies in this age group are crucial because this is the time when the first symptoms of these disorders appear and when significant neuroplastic processes occur. Therefore, the purpose of the present study was to assess the serum levels of NPY and PYY in adolescents with OB or AN, as well as in a healthy control group (CG). A secondary goal was to test for the effects of potential confounders on the concentrations of NPY and PYY, including age, body mass index (BMI), and glucose and insulin levels, and to explore the impact of eating disorders and depressive symptoms on these neuropeptides in adolescents across the weight spectrum. We expected that the concentrations of NPY and PYY might be associated with homeostatic regulation factors such as glucose or insulin, and also with the emotions, attitudes, and behaviours connected with food intake.

## 2. Methodology

### 2.1. Participants and Procedures

Eighty-nine adolescents (11 boys and 78 girls) were included in the study. Thirty of them were admitted to the Department of Pediatric Diabetes and Obesity because of obesity (OB group). Thirty girls were admitted in the acute phase of anorexia nervosa (AN group) to the Department of Child and Adolescent Psychiatry. The remaining 29 subjects were recruited among middle school students (CG). Patients with AN were hospitalised for the first time and had symptoms of the disease for less than a year. Diagnosis of the restrictive type of AN was made according to the 10th revision of International Statistical Classification of Diseases and Related Health Problems (ICD-10) and 5th revision of Diagnostic and Statistical Manual of Mental Disorders (DSM-5). The inclusion criteria for AN and OB were age- and gender-adjusted BMI percentile of 5 and 95, respectively. The exclusion criteria for all participants, including CG, were the occurrence of any psychiatric disorders based on the interview conducted by a specialist in child and adolescent psychiatry, chronic somatic illnesses and laboratory abnormalities not related to OB or AN, chronic medication use, and dietary supplement intake.

All patients had the following checks within two days of admission: Height and weight were checked, psychometric evaluation was carried out, and 15 mL of blood was obtained. BMI was calculated as the ratio of body weight (kg) to height squared (m^2^), and the percentage of ideal body weight (%IBW) as a ratio of actual to ideal body weight (IBW) × 100%, where IBW (kg) = height (cm) − 100 − {[(height (cm) − 150)]/2} according to Lorentz’s formula [44].

The Beck Depression Inventory (BDI), the Body Attitude Test (BAT), and the Eating Attitudes Test (EAT-26) have high reliability and validity and are widely applied to assesses specific psychopathological symptoms in adolescents [45,46,47,48,49]; therefore, they were used in the present study. The BDI is a 21-item self-report inventory used for measuring the symptoms of depression. It evaluates mood, pessimism, sense of failure, self-dissatisfaction, guilt, punishment, self-dislike, self-accusation, suicidal ideas, crying, irritability, social withdrawal, indecisiveness, body image change, work difficulty, insomnia, fatigability, loss of appetite, weight loss, somatic preoccupation, and loss of libido. The standard cut-off scores are as follows: 0–11 without depression, 12–26 mild depression, 27–49 moderately severe depression, and 50–63 very severe depression [50]. BAT is a self-assessment questionnaire designed to assess subjective body experience and attitude towards one’s own body. The items are divided into the following three subscales: (1) negative appreciation of body size, with questions concerning dissatisfaction with body size and its specific parts; (2) lack of familiarity with one’s own body, with questions related to body perception and feelings of tension, numbness, alienation, and danger; (3) general dissatisfaction, with questions concerning general dissatisfaction, controlling and comparing one’s appearance [51]. The EAT-26 is a standardised measurement of symptoms and fears characteristic of EDs. It is a self-report questionnaire that consists of 26 items over the following three subscales: (1) dieting, including questions about reducing food intake and the emotions accompanying it; (2) bulimia and food preoccupation, with questions related to time spent eating, binge eating, and vomiting episodes; (3) oral control, with questions pertaining to overeating control [52]. Scores over 20 indicate the possibility of ED. Moreover, in all used tests, the higher the score, the greater the severity of the measured symptoms.

The CG also underwent a physical and mental examination, anthropometric measurements, psychological assessment (except the BAT), and laboratory tests. The research protocol was approved by the Poznan University of Medical Sciences Bioethics Committee (265/18 and 295/18). All procedures were conducted according to the 1964 Declaration of Helsinki. Consent was obtained from all participants and their guardians.

### 2.2. Biochemical Analysis

Venous blood was collected on morning admission (8:00–9:00 a.m.) from fasting (12 h after the last meal) AN, OB, and CG subjects. Serum was immediately separated from the blood by centrifugation at 1000× *g* for 15 min at 4 °C, aliquoted into Eppendorf tubes, frozen at −70 °C, and assayed afterwards.

Quantitative NPY and PYY tests were performed using a commercial one enzyme-linked immunosorbent assay (Human Pro-neuropeptide Y Elisa Kit, cat. No. E0879h, and Human Peptide YY Elisa Kit, cat. No. E1067h), following the manufacturer’s instructions.

The measurement range of the kit was 78–5000 pg/mL for NPY and 6.25–400 pg/mL for PYY. The minimum detectable dose of general NPY is typically less than 32 pg/mL, and for PYY, less than 3.1 pg/mL. Optical density was read via a spectrophotometric plate reader (Biochrom Asys UVM 340 Microplate Reader) at a wavelength of 450 ± 10 nm. Every assay was performed twice, and the mean value of the two assays was used for statistical evaluation. A four-parameter algorithm (four-parameter logistic) was used to assay the concentration in the tested samples.

### 2.3. Statistical Analysis

The analyses were carried out in the PQStat program, version 1.8.0.352. The data are described as arithmetic means ± standard deviation (SD). The results are also presented as boxplots.

The normality of the distribution was tested by the Shapiro–Wilk test. When the data distribution differed significantly from the normal distribution, the groups were compared by the Kruskal–Wallis test and Dunn’s multiple comparison test with Bonferroni correction, or by the Mann–Whitney U test, depending on the number of compared groups. For variables with a normal distribution, the ANOVA test for independent variables and Tukey’s HSD multiple comparison test were used. The effect sizes for each comparison were also calculated.

The impact of potential confounding variables on the NPY and PYY levels were analysed in all included adolescents across the weight spectrum (AN + OB + CG). For this purpose, log-transformation of the analysed variables was conducted due to the presence of outliers and non-normal distributions. However, the data remained non-normal for most of the variables. Therefore, generalised linear models were used to perform the analysis. A significance level of 0.05 was used for the statistical analyses.

## 3. Results

### 3.1. Demographic Characteristics

The demographic characteristics of all participants are presented in Table 1. The study groups showed statistically significant age differences (*p* < 0.001). The OB group was statistically significantly younger than the CG and the AN group; however, there was no difference between the CG and the AN group. As expected, the AN and OB groups and the CG differed significantly in terms of BMI (*p* < 0.001) and percentage of ideal body mass (%IBW) (*p* < 0.001).

### 3.2. Levels of Neuropeptide Y (NPY) and Peptide YY (PYY)

**Figure 1:**
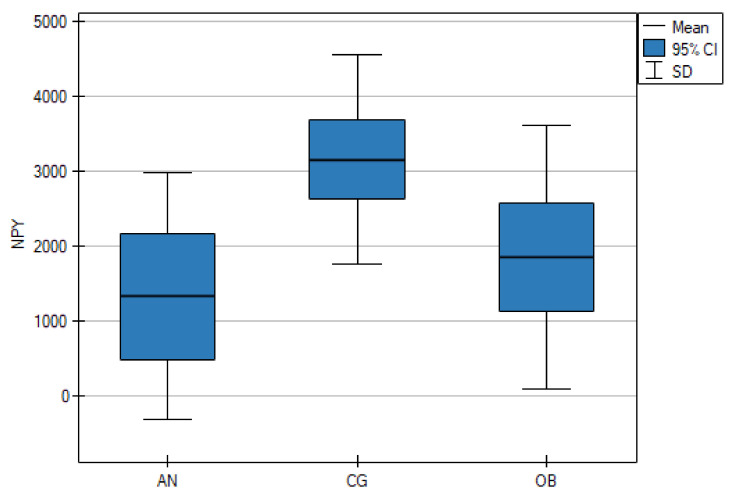
Fasting serum levels of neuropeptide Y (NPY) in adolescents with anorexia nervosa (AN) or obesity (OB), as well as in the healthy control group (CG).

**Figure 2:**
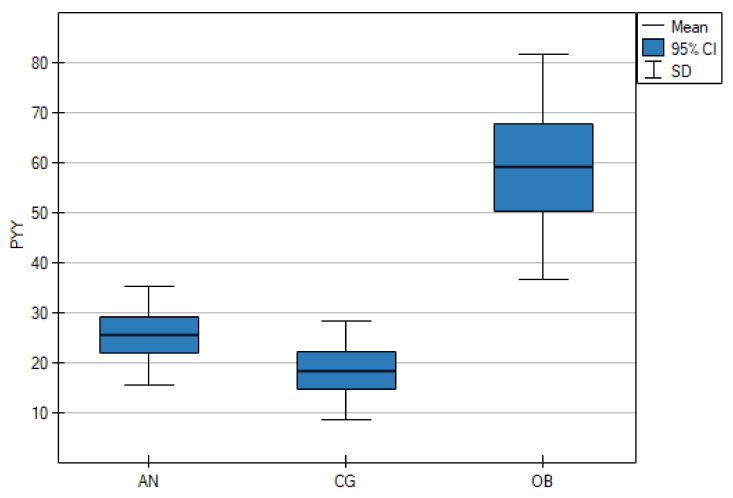
Fasting serum levels of peptide YY (PYY) in adolescents with anorexia nervosa (AN) or obesity (OB), as well as in the healthy control group (CG).

As shown in Table 1, the serum NPY concentrations were significantly lower in the AN group than in the CG (*p* < 0.001), but there were no statistically significant differences between the AN and OB groups (*p* = 0.661). However, the OB group and the CG differed significantly (*p* = 0.006) (Figure 1). The mean PYY concentrations in the AN group and the CG were lower than in the OB group (*p* < 0.001) (Figure 2).

### 3.3. Clinical Characteristics

As presented in Table 2, the AN group obtained significantly higher results in the BDI than the CG (*p* < 0.001) and the OB group (*p* = 0.040), but there was no statistically significant difference between the OB group and the CG (*p* = 0.205). Patients with OB obtained statistically higher total BAT scores than the AN group (*p* = 0.010). They also differed in two subscales, i.e., the negative appreciation of body size (*p* = 0.003) and the lack of familiarity with one’s own body (*p* = 0.028). There were no significant differences in the total EAT-26 score among the AN and OB group and the CG (AN group vs. CG, *p* = 0.163; AN group vs. OB group, *p* = 1; OB group vs. CG, *p* = 0.075). However, on the dieting subscale, there were significant differences both between the OB and AN groups (*p* < 0.001) and between the OB group and the CG (*p* < 0.001). Furthermore, in the oral control subscale, there was a significant difference between the AN and OB groups (*p* < 0.022).

### 3.4. Analysis of Confounding Variables

The findings of the regressions assessing age, BMI, and fasting serum glucose and insulin, as well as the selected psychopathological symptoms, as potential confounders of the fasting serum concentrations of NPY and PYY in adolescents across the weight spectrum are shown in Table 3. The severity of eating disorder symptoms measured with the EAT-26 predicted fasting serum concentrations of NPY (*p* = 0.012) but not PYY. However, age, BMI, and serum fasting glucose and insulin levels were not associated with NPY or PYY.

## 4. Discussion

Patients with OB or AN and healthy subjects under the age of 18 were enrolled in the present study. They did not differ in height, but because of their diagnoses, they differed in body weight, BMI, and percentage of ideal body weight. Furthermore, they differed in the intensity of some psychopathological symptoms. Patients diagnosed with AN presented significantly higher results in the BDI than the CG and the OB group, which is congruent with reports that up to 70% of patients with AN have comorbid depressive symptoms.

Subjects with OB scored higher than the AN group in the BAT scale, and the differences were mainly in two subscales, i.e., negative appreciation of body size and lack of familiarity with one’s own body. The higher results in the BAT in the OB group could be influenced by the devaluation and social stigmatisation of obesity [53,54], which should be considered when planning weight loss interventions. However, these findings were interesting regarding patients with AN, since they are widely believed to be dissatisfied with their appearance. Such low BAT scores among patients with AN might be related to the fact that at the time of testing, they had an extremely low body weight, which could be satisfactory for them.

However, it is more likely that AN patients’ self-assessment results were influenced by the denial of the disease symptoms, as reported in previous studies [55,56]. Therefore, the results of the EAT-26 scale were also not as high as expected. Although the mean results for the total scores on the EAT-26 scale for the AN group were 14.07, for the CG were 5.17, and for the OB group were 11.07, the level of statistical significance was not reached (*p* = 0.059). However, significant differences were observed in the subscales. Patients with AN were characterised by a greater intensity of oral control symptoms, and OB subjects by greater intensity of diet symptoms, which is congruent with the clinical observations and previous studies [57,58]. Thus, we suggest that good practice in future studies of patients with AN would be to not only use self-assessment scales, but also those assessed by a specialist.

Numerous studies have shown that NPY levels are rising due to a lack of food, which, in turn, leads to maintaining the homeostatic balance by reducing energy expenditure, stimulating appetite, and increasing food intake [59,60,61]. NPY, as an orexigenic peptide, performs several functions in the regulation of appetite and energy homeostasis. It delays the appearance of satiety, increases the size of the meal, extends the time of eating [62,63,64,65,66], stimulates carbohydrate preference, and affects the choice of food [27].

The adolescent patients with increased body weight included in the present study had reduced fasting serum NPY levels as compared with healthy individuals. These results are congruent with the studies conducted by Saranck and Lou [67,68] among obese children. To the best of our knowledge, there is no similar research in children or adolescents. However, in adults with increased body weight, the NPY serum levels were reported to be higher than in healthy subjects [69,70,71] or remained unchanged [72,73]. We speculate that the differences in the results may be related to the age of the respondents, especially since animal studies have shown that the level of NPY expression is age dependent and increases with age. Additionally, it should be investigated whether the duration of obesity affects the differences in peptide alteration.

However, the reduced serum NPY levels in the recruited OB patients could be an adaptive mechanism for increased food intake. Furthermore, this effect may be mediated by leptin, which inhibits NPY and is elevated in obesity. Therefore, it seems necessary to conduct research that could explain the causes of this phenomenon.

The adolescent patients with decreased body weight had also reduced fasting serum NPY levels as compared with healthy individuals. Previous studies have shown conflicting results, for example, NPY concentrations have been reported to be decreased [74], increased [75,76,77], or unchanged [71,78]. However, only one of these studies was conducted in adolescents. The decreased NPY levels in malnourished patients with AN suggests no NPY response to starvation, which could be relevant for maintaining a negative energy balance and upholding the disease symptoms. Moreover, it has been proven that the elevation of NPY reduces anxiety, fear learning, and locomotor activity [79]. Therefore, the authors of the present study suggest that the increased anxiety and physical activity observed in patients with AN might be partially associated with a decreased level of NPY. It would be worth continuing research in this area.

PYY is a potent anorexigenic agent. It has been suggested that peripheral PYY acts as a satiety signal, regulating the termination of individual meals, partly by reducing the production of the hunger-stimulating peptide ghrelin. The results obtained in the patients with AN confirm previous reports, i.e., the serum fasting PYY concentrations were not significantly different from those of the healthy volunteers [77,80,81,82]. However, some other studies have reported an increase [83]. We expected PYY to be lower in malnourished patients; however, it remained unchanged as compared with the control group. The present study did not explain whether the lack of changes in the PPY concentration is related to the etiopathogenesis of the disorder, as Pfluger suggested [84], or whether PPY is not crucial for AN. These questions indicate the need for further research.

The fasting serum PYY levels were higher in OB adolescents, which is congruent with the research conducted by Patel [85]. However, in most studies among obese youths, their fasting PYY levels were in a normal range [86,87,88,89,90] or lower [91,92]. Equivocal results have been obtained in adults. The PYY was usually decreased [12,81,93,94] or remained unchanged [82,95,96,97]. A higher level of PYY in obesity is adequate for metabolic status, although it has no effect. It should be considered whether this is related to PYY resistance, which is also observed in other anorexigenic peptides such as leptin [98]. However, most of the studies on adult patients do not support PPY resistance in individuals with obesity [99]. Unfortunately, the present research did not explain the differences in the results of previous studies regarding PYY. It has already been reported that the role of appetite-related gut peptides in childhood and adolescent obesity is less explored and less understood, and findings can be challenging to interpret in this age group [100]. Puberty and body composition, which change with age, are the crucial factors affecting the peptides involved in food intake regulation. Therefore, comparing the results of similar studies in children and adults might not be reliable. Thus, it is necessary to conduct more research on the proteins regulating obese youths’ metabolism, because they may act differently than in adults.

The severity of eating disorder symptoms measured with the EAT-26 scale predicted the concentration of NPY in adolescents across the weight spectrum. The neural neuropeptide Y receptor system might be involved in the processes connected with eating, because the localisation of its receptors overlaps with the neural networks engaged in cue-conditioned eating in limbic and cortical areas [101]. To the best of our knowledge, there have been no studies investigating the correlation between NPY and eating disorder symptomatology; therefore, it seems necessary to continue research in this area.

The severity of depression symptoms measured with the BDI scale did not predict the NPY concentration in all of the included participants, although the results were at the borderline of statistical significance (*p* = 0.059). In previous studies, adolescents with higher scores in BDI had lower plasma neuropeptide Y levels. The NPY receptors in the hippocampus, cerebral cortex, and amygdala are essential for NPY regulation of anxiety, reactivity to stress, cognitive processes, and depression [102]. Animal studies have suggested that lower NPY levels are associated with posttraumatic stress disorder (PTSD), depression, and anxiety-like symptoms [37], and intracerebral NPY administration reduces anxiety and improves mood [30,103]. A meta-analysis of the studies conducted among patients with major depressive disorder (MDD) confirmed that NPY levels are decreased in this disease [104]. It has been postulated that the regulation of central NPY expression is one of the mechanisms of adaptation to chronic stress, which is reflected in the pathophysiology of anxiety and depression. Many reports have indicated that the Y1 receptor primarily mediates the anxiolytic activity of NPY; An intra-amygdala injection of NPY increases social interaction time and has an anxiolytic-like effect [36,105]. NPY adapts the body to stressful, potentially life-threatening conditions and maintains resistance to traumatic events and psychological integrity [106]. Therefore, research in this area should be continued.

NPY and PYY were not related to age, BMI, or fasting serum of insulin and glucose in adolescents across the weight spectrum. This is congruent with many previous studies conducted among healthy people, as well as those suffering from some somatic diseases [95,107,108,109]. Since BMI reflects the degree of nutrition, and glucose and insulin provide this information to the relevant brain areas, a correlation between these variables and NPY and PYY might be expected. However, associations between metabolic status and appetite-regulating peptides are more complex than simple feedback loops [110], and the higher regions of the brain connected with emotions and behaviours might be important in their regulation.

The authors of the present study are aware of its limitations and recall the most important of them. The number of considered variables was quite large, which reduced the power of each analysis. The healthy controls were not issued the Body Attitude Test. However, we compared the results between the AN and OB groups, and since they seemed interesting, we considered presenting and discussing them. Moreover, we did not use BAT in the confounding variables analysis. The subjects included in the study were instructed to eat their last meal 12 h before the test. We did not use any additional methods to validate the fasting state, which may have diluted the findings. However, they were also not used in the above discussed research. While interpreting the results of this study, it should be remembered that the level of peripheral peptides does not have to reflect their central activity. Moreover, the aetiology of anorexia nervosa and obesity is complex, and only single factors in the entire complex network of relationships were analysed. Furthermore, it is difficult to determine which of the alterations in protein levels are primary and which are secondary to the changes in body weight, as well as what their final role in etiopathogenesis is. Therefore, continued follow-up studies on a larger group of adolescents with AN and OB after body weight normalisation are strongly recommended. It would also be valuable to compare NPY and PYY levels across the whole spectrum of ED, including patients with bulimia nervosa and binge eating disorders. In OB and AN, circulating protein levels could be decreased, which should be considered in further analysis.

## 5. Conclusions

The fasting serum levels of neuropeptide Y were decreased in adolescent patients with anorexia nervosa, as well as in those with obesity. This suggests the need to look for a common link in the mechanism of this effect, despite the opposite metabolic status in such different groups of patients. The right direction of further research should be taking into consideration additional confounding variables, including those related to the mental state of the patients.

Adolescent patients with obesity, but not with anorexia nervosa or healthy patients, had increased fasting serum concentrations of peptide YY. This is interesting, especially since other gastrointestinal peptides are also altered in this condition and are used in the treatment; therefore, more studies on PYY are needed.

Correlations between the centrally produced neuropeptide Y and the severity of eating disorder symptoms require further study in this area. We suggest that psychopathological symptoms have an influence on the neurohormones regulating metabolism. This might be crucial for therapeutic interventions in both anorexia and obesity.

## Figures and Tables

**Table 1:** Age, height, body mass index (BMI), percentage of ideal body mass (% IBW), and fasting serum levels of glucose, insulin, neuropeptide Y (NPY), and peptide YY (PYY) in adolescents with anorexia nervosa (AN) or obesity (OB), as well as in the healthy control group (CG).

	*N*	AN	*N*	CG	*N*	OB	*p*	*p* Multiple Comparisons			η^2^
								AN vs. CG	AN vs. OB	CG vs. OB	
Age (years)	30	14.83 ± 1.74	29	15.38 ± 1.47	30	12.47 ± 3.26	<0.001	1	0.005	<0.001	0.190
Height (m)	30	1.61 ± 0.07	29	1.65 ± 0.05	30	1.61 ± 0.09	0.147	0.091	0.098	0.088	0.043
BMI (kg/m^2^)	30	14.07 ± 1.38	29	19.14 ± 5.32	30	32.46 ± 5.98	<0.001	<0.001	<0.001	<0.001	0.803
%IBW	29	0.63 ± 0.15	29	0.94 ± 0.18	30	1.53 ± 0.3	<0.001	<0.001	<0.001	<0.001	0.814
Glucose (mg/dL)	20	76.2 ± 7.47	26	77.38 ± 7.53	30	89.33 ± 6.16	<0.001	0.083	<0.001	<0.001	0.442
Insulin (mU/mL)	16	8.02 ± 10.19	26	9.81 ± 7.27	30	19.69 ± 7.26	<0.001	0.444	<0.001	<0.001	0.446
NPY (pg/mL)	27	1328.69 ± 1647.57	30	3159.69 ± 1401.19	25	1850.03 ± 1769.06	<0.001	0.001	0.661	<0.006	0.242
PYY (pg/mL)	30	25.49 ± 9.79	28	18.46 ± 9.81	28	59.12 ± 22.5	<0.001	0.084	<0.001	<0.001	0.563

Data are presented as mean ± standard deviation.

**Table 2:** The Beck Depression Inventory (BDI), the Body Attitude Test (BAT), and the Eating Attitude Test (EAT-26) scores in adolescents with anorexia nervosa (AN) or obesity (OB), as well as in the healthy control group (CG).

	*N*	AN	*N*	CG	*N*	OB	*p*	*p* Multiple Comparisons			η^2^
								AN vs. CG	AN vs. OB	CG vs. OB	
BDI	26	21.00 ± 14.53	30	7.7 ± 8.26	29	11.86 ± 9.75	<0.001	<0.001	0.040	0.205	0.199
BAT total score	16	21.13 ± 22.73	0	-	30	38.97 ± 18.95	0.010	-	-	-	0.652
BAT negative appreciation of body size	16	7.75 ± 9.73	0	-	30	17.27 ± 8.98	0.003	-	-	-	0.650
BAT lack of familiarity with one’s own body	16	7.31 ± 7.42	0	-	30	12 ± 6.01	0.028	-	-	-	0.654
BAT general dissatisfaction	16	6.06 ± 6.39	0	-	30	9.7 ± 5.52	0.056	-	-	-	0.655
EAT-26 total score	30	14.03 ± 16.43	30	5.17 ± 5.6	30	11.07 ± 12.3	0.053	0.163	1	0.075	0.043
EAT-26 dieting	30	2.41 ± 3.61	30	2.33 ± 3.58	30	7.83 ± 7.46	<0.001	1	<0.001	<0.001	0.245
EAT-26 bulimia and food preoccupation	30	2.09 ± 3.55	30	0.7 ± 1.39	30	1.47 ± 2.73	0.530	0.800	1	1	0.008
EAT-26 oral control	30	4.63 ± 5.19	30	2.2 ± 2.96	30	1.77 ± 3.59	0.024	0.268	0.022	0.993	0.061

Data are presented as mean ± standard deviation.

**Table 3:** Age, BMI, fasting serum glucose and insulin, and psychopathological symptoms measured with the Eating Attitudes Test (EAT-26) and the Beck Depression Inventory (BDI) as the potential confounders of neuropeptide Y (NPY) and peptide YY (PYY) levels in adolescents across the weight spectrum (anorexia nervosa, obesity, and healthy control group).

	NPY			PYY		
	*N*	B	*p*	*N*	B	*P*
Age (years)	70	−0.09	0.432	84	−0.06	0.316
BMI (kg/m^2^)	69	0.11	0.422	83	0.06	0.705
Glucose (mg/dL)	61	0.40	0.227	71	0.19	0.683
Insulin (mU/mL)	59	−0.04	0.559	67	0.11	0.287
BDI	61	−0.06	0.059	76	−0.03	0.460
EAT-26	58	−0.08	0.002	72	0.02	0.645

## References

[1] Haines J., Kleinman K.P., Rifas-Shiman S.L., Field A.E., Austin S.B. (2010). Examination of shared risk and protective factors for overweight and disordered eating among adolescents. Arch. Pediatr. Adolesc. Med..

[2] Jacobi C., Hayward C., De Zwaan M., Kraemer H.C., Agras W.S. (2004). Coming to terms with risk factors for eating disorders: Application of risk terminology and suggestions for a general taxonomy. Psychol. Bull..

[3] Faulconbridge L.F., Bechtel C.F. (2014). Depression and disordered eating in the obese person. Curr. Obes. Rep..

[4] Inui A., Meguid M.M. (2003). Cachexia and obesity: Two sides of one coin?. Curr. Opin. Clin. Nutr. Metab. Care.

[5] Tsai V.W.W., Lin S., Brown D.A., Salis A., Breit S.N. (2015). Anorexia–cachexia and obesity treatment may be two sides of the same coin: Role of the TGF-b superfamily cytokine MIC-1/GDF15. Int. J. Obes..

[6] Day J., Ternouth A., Collier D.A. (2009). Eating disorders and obesity: Two sides of the same coin?. Epidemiol. Psichiatr. Soc..

[7] Treasure J., Zipfel S., Micali N., Wade T., Stice E., Claudino A., Schmidt U., Frank G.K., Bulik C.M., Wentz E. (2015). Anorexia nervosa. Nat. Rev. Dis. Primers.

[8] Bulik C.M., Flatt R., Abbaspour A., Carroll I. (2019). Reconceptualizing anorexia nervosa. Psychiatry Clin. Neurosci..

[9] Watson H.J., Initiative A.N.G., Yilmaz Z., Thornton L.M., Hübel C., Coleman J.R.I., Gaspar H.A., Bryois J., Hinney A., Leppä V.M. (2019). Genome-wide association study identifies eight risk loci and implicates metabo-psychiatric origins for anorexia nervosa. Nat. Genet..

[10] Chaudhri O.B., Field B.C.T., Bloom S.R. (2006). From gut to mind—Hormonal satiety signals and anorexia nervosa. J. Clin. Endocrinol. Metab..

[11] Williams D.L., Baskin D.G., Schwartz M.W. (2006). Leptin regulation of the anorexic response to glucagon-like peptide-1 receptor stimulation. Diabetes.

[12] Yau Y.H.C., Potenza M.N. (2013). Stress and eating behaviors. Minerva Endocrinol..

[13] Cox H.M. (2007). Peptide YY: A neuroendocrine neighbor of note. Peptides.

[14] Cox H.M. (2007). Neuropeptide Y receptors; antisecretory control of intestinal epithelial function. Auton. Neurosci..

[15] Ekblad E., Sundler F. (2002). Distribution of pancreatic polypeptide and peptide YY. Peptides.

[16] McGowan B.M.C., Bloom S.R. (2004). Peptide YY and appetite control. Curr. Opin. Pharmacol..

[17] Adrian T., Ferri G.-L., Bacarese-Hamilton A., Fuessl H., Polak J., Bloom S. (1985). Human distribution and release of a putative new gut hormone, peptide YY. Gastroenterology.

[18] Batterham R.L., Cohen M.A., Ellis S.M., Le Roux C.W., Withers D.J., Frost G.S., Ghatei M.A., Bloom S.R. (2003). Inhibition of food intake in obese subjects by peptide YY3–36. N. Engl. J. Med..

[19] Spreckley E., Murphy K.G. (2015). The L-cell in nutritional sensing and the regulation of appetite. Front. Nutr..

[20] Gustafson E.L., Smith K.E., Durkin M.M., Walker M.W., Gerald C., Weinshank R., Branchek T.A. (1997). Distribution of the neuropeptide Y Y2 receptor mRNA in rat central nervous system. Mol. Brain Res..

[21] Dumont Y., Jacques D., Bouchard P., Quirion R. (1998). Species differences in the expression and distribution of the neuropeptide Y Y1, Y2, Y4, and Y5 receptors in rodents, guinea pig, and primates brains. J. Comp. Neurol..

[22] Eaton K., Sallee F.R., Sah R. (2007). Relevance of neuropeptide Y (NPY) in psychiatry. Curr. Top. Med. Chem..

[23] Kask A., Harro J., Von Hörsten S., Redrobe J.P., Dumont Y., Quirion R. (2002). The neurocircuitry and receptor subtypes mediating anxiolytic-like effects of neuropeptide Y. Neurosci. Biobehav. Rev..

[24] Wettstein J., Earley B., Junien J. (1995). Central nervous system pharmacology of neuropeptide Y. Pharmacol. Ther..

[25] Nergårdh R., Ammar A., Brodin U., Bergström J., Scheurink A., Södersten P. (2007). Neuropeptide Y facilitates activity-based-anorexia. Psychoneuroendocrinology.

[26] Steinman J.L., Gunion M.W., Morley J.E. (1994). Forebrain and hindbrain involvement of neuropeptide Y in ingestive behaviors of rats. Pharmacol. Biochem. Behav..

[27] Wang J., Akabayashi A., Dourmashkin J., Yu H.J., Alexander J.T., Chae H.J., Leibowitz S.F. (1998). Neuropeptide Y in relation to carbohydrate intake, corticosterone and dietary obesity. Brain Res..

[28] Jang M., Mistry A., Swick A.G., Romsos D.R. (2000). Leptin rapidly inhibits hypothalamic neuropeptide Y secretion and stimulates corticotropin-releasing hormone secretion in adrenalectomized mice. J. Nutr..

[29] Schéle E., Grahnemo L., Anesten F., Hallén A., Bäckhed F., Jansson J.-O. (2013). The gut microbiota reduces leptin sensitivity and the expression of the obesity-suppressing neuropeptides proglucagon (Gcg) and brain-derived neurotrophic factor (Bdnf) in the central nervous system. Endocrinology.

[30] Edelsbrunner M., Painsipp E., Herzog H., Holzer P. (2009). Evidence from knockout mice for distinct implications of neuropeptide-Y Y2 and Y4 receptors in the circadian control of locomotion, exploration, water and food intake. Neuropeptides.

[31] Edelsbrunner M.E., Herzog H., Holzer P. (2009). Evidence from knockout mice that peptide YY and neuropeptide Y enforce murine locomotion, exploration and ingestive behaviour in a circadian cycle- and gender-dependent manner. Behav. Brain Res..

[32] Zhang L., Nguyen A.D., Lee I.-C.J., Yulyaningsih E., Riepler S.J., Stehrer B., Enriquez R.F., Lin S., Shi Y.-C., Baldock P.A. (2012). NPY modulates PYY function in the regulation of energy balance and glucose homeostasis. Diabetes Obes. Metab..

[33] Heilig M. (2004). The NPY system in stress, anxiety and depression. Neuropeptides.

[34] Holzer P., Reichmann F., Farzi A. (2012). Neuropeptide Y, peptide YY and pancreatic polypeptide in the gut–brain axis. Neuropeptides.

[35] Morales-Medina J.C., Dumont Y., Quirion R. (2010). A possible role of neuropeptide Y in depression and stress. Brain Res..

[36] Sajdyk T.J., Vandergriff M., Gehlert D.R. (1999). Amygdalar neuropeptide Y Y1 receptors mediate the anxiolytic-like actions of neuropeptide Y in the social interaction test. Eur. J. Pharmacol..

[37] Stogner K.A., Holmes P.V. (2000). Neuropeptide-Y exerts antidepressant-like effects in the forced swim test in rats. Eur. J. Pharmacol..

[38] Bannon A., Seda J., Carmouche M., Francis J., Norman M., Karbon B., McCaleb M. (2000). Behavioral characterization of neuropeptide Y knockout mice. Brain Res..

[39] Painsipp E., Herzog H., Sperk G., Holzer P. (2011). Sex-dependent control of murine emotional-affective behaviour in health and colitis by peptide YY and neuropeptide Y. Br. J. Pharmacol..

[40] Yamada C., Mogami S., Kanno H., Hattori T. (2018). Peptide YY causes apathy-like behavior via the dopamine D2 receptor in repeated water-immersed mice. Mol. Neurobiol..

[41] Corvino V., Marchese E., Podda M.V., Lattanzi W., Giannetti S., Di Maria V., Cocco S., Grassi C., Michetti F., Geloso M.C. (2014). The neurogenic effects of exogenous neuropeptide y: Early molecular events and long-lasting effects in the hippocampus of trimethyltin-treated rats. PLoS ONE.

[42] Lin X., Qi Q., Zheng Y., Huang T., Lathrop M., Zelenika D., Bray G.A., Sacks F.M., Liang L., Qi L. (2015). Neuropeptide Y genotype, central obesity, and abdominal fat distribution: The POUNDS LOST trial 1,2. Am. J. Clin. Nutr..

[43] Cahill F., Ji Y., Wadden D., Amini P., Randell E., Vasdev S., Gulliver W., Sun G. (2014). The association of serum total peptide YY (PYY) with obesity and body fat measures in the CODING study. PLoS ONE.

[44] Nahler G. (2009). Lorentz-Formula. Dictionary of Pharmaceutical Medicine.

[45] Rivas T., Bersabé R., Jiménez M., Berrocal C. (2010). The eating attitudes test (EAT-26): Reliability and validity in Spanish female samples. Span. J. Psychol..

[46] Rogoza R., Brytek-Matera A., Garner D. (2016). Analysis of the EAT-26 in a non-clinical sample. Arch. Psychiatry Psychother..

[47] Brytek-Matera A., Probst M. (2014). Psychometric properties of the Polish version of the Body Attitude Test. Arch. Psychiatry Psychother..

[48] Stockings E., Degenhardt L., Lee Y.Y., Mihalopoulos C., Liu A., Hobbs M., Patton G. (2015). Symptom screening scales for detecting major depressive disorder in children and adolescents: A systematic review and meta-analysis of reliability, validity and diagnostic utility. J. Affect. Disord..

[49] Gila A., Castro J., Gómez M.J., Toro J., Salamero M. (1999). The body attitude test: Validation of the Spanish version. Eat. Weight Disord. Stud. Anorex. Bulim. Obes..

[50] Beck A.T., Ward C.H., Mendelson M., Mock J., Erbaugh J. (1961). An inventory for measuring depression. Arch. Gen. Psychiatry.

[51] Probst M., Vandereycken W., Van Coppenolle H., Vanderlinden J. (1995). The body attitude test for patients with an eating disorder: Psychometric characteristics of a new questionnaire. Eat. Disord..

[52] Garner D.M., Olmstead M.P., Polivy J. (1983). Development and validation of a multidimensional eating disorder inventory for anorexia nervosa and bulimia. Int. J. Eat. Disord..

[53] Di Pasquale R., Celsi L. (2017). Stigmatization of overweight and obese peers among children. Front. Psychol..

[54] Schwimmer J.B., Burwinkle T.M., Varni J.W. (2003). Health-related quality of life of severely obese children and adolescents. JAMA.

[55] Couturier J., Lock J. (2006). Denial and minimization in adolescents with anorexia nervosa. Int. J. Eat. Disord..

[56] Viglione V., Muratori F., Maestro S., Brunori E., Picchi L. (2006). Denial of symptoms and psychopathology in adolescent anorexia nervosa. Psychopathology.

[57] Orbitello B., Ciano R.P., Corsaro M., Rocco P.L., Taboga C., Tonutti L., Armellini M., Balestrieri M. (2006). The EAT-26 as screening instrument for clinical nutrition unit attenders. Int. J. Obes..

[58] Alkazemi D.U., Zafar T.A., Ebrahim M., Kubow S. (2018). Distorted weight perception correlates with disordered eating attitudes in Kuwaiti college women. Int. J. Eat. Disord..

[59] Flier J.S. (2004). Obesity wars. Cell.

[60] Sainsbury A., Zhang L. (2010). Role of the arcuate nucleus of the hypothalamus in regulation of body weight during energy deficit. Mol. Cell. Endocrinol..

[61] Shi Y.-C., Lau J., Lin Z., Zhang H., Zhai L., Sperk G., Heilbronn R., Mietzsch M., Weger S., Huang X.-F. (2013). Arcuate NPY controls sympathetic output and BAT function via a relay of tyrosine hydroxylase neurons in the PVN. Cell Metab..

[62] Leibowitz S., Alexander J. (1991). Analysis of neuropeptide Y-induced feeding: Dissociation of Y1 and Y2 receptor effects on natural meal patterns. Peptides.

[63] Lynch W.C., Hart P., Babcock A.M. (1994). Neuropeptide Y attenuates satiety: Evidence from a detailed analysis patterns ingestion. Brain Res..

[64] Stricker-Krongrad A., Max J., Musse N., Nicolas J., Burlet C., Beck B. (1994). Increased threshold concentrations of neuropeptide Y for a stimulatory effect on food intake in obese Zucker rats—Changes in the microstructure of the feeding behavior. Brain Res..

[65] Yang K., Guan H., Arany E., Hill D.J., Cao X. (2008). Neuropeptide Y is produced in visceral adipose tissue and promotes proliferation of adipocyte precursor cells via the Y1 receptor. FASEB J..

[66] Rowland N.E. (1988). Peripheral and central satiety factors in neuropeptide Y-induced feeding in rats. Peptides.

[67] Saranac L., Bjelakovic B., Stamenković H., Kamenov B. (2007). Orexitropic signaling proteins in obese children. Sci. World J..

[68] Lou X.-M., Duan G.-C., Chen J., Zhou Y., Hu Q.-Y., Heng Z.-C. (2006). Blood leptin, orexins and NPY levels and their relations in obese children. Sichuan Da Xue Xue Bao Yi Xue Ban.

[69] Baltazi M., Katsiki N., Savopoulos C., Iliadis F., Koliakos G., Hatzitolios A.I. (2011). Plasma neuropeptide Y (NPY) and alpha-melanocyte stimulating hormone (a-MSH) levels in patients with or without hypertension and/or obesity: A pilot study. Am. J. Cardiovasc. Dis..

[70] Baranowska B., Wolińska-Witort E., Martyńska M., Chmielowska M., Baranowska-Bik A. (2005). Plasma orexin A, orexin B, leptin, neuropeptide Y (NPY) and insulin in obese women. Neuro Endocrinol. Lett..

[71] Baranowska B., Wasilewska-Dziubińska E., Radzikowska M., Pęlonowski A., Roguski K. (1997). Neuropeptide Y, galanin, and leptin release in obese women and in women with anorexia nervosa. Metabolism.

[72] Nam S.-Y. (2001). Cerebrospinal fluid and plasma concentrations of leptin, NPY, and -MSH in obese women and their relationship to negative energy balance. J. Clin. Endocrinol. Metab..

[73] Milewicz A., Bidzinska B., Mikulski E., Demissie M., Tworowska U. (2000). Influence of obesity and menopausal status on serum leptin, cholecystokinin, galanin and neuropeptide Y levels. Gynecol. Endocrinol..

[74] Baranowska B., Wolinska-Witort E., Wasilewska-Dziubinska E., Roguski K., Chmielowska M. (2001). Plasma leptin, neuropeptide Y (NPY) and galanin concentrations in bulimia nervosa and in anorexia nervosa. Neuro Endocrinol. Lett..

[75] Jagielska G., Bartoszewicz Z., Niedźwiedzka B., Kondracka A., Brzozowska A., Karowicz-Bilińska A. (2013). Assessment of neuropeptide Y, leptin and leptin-receptor concentrations in teenagers suffering from anorexia nervosa. Ginekol. Polska.

[76] Sedláčková D., Kopečková J., Papežová H., Vybíral S., Kvasničková H., Hill M., Nedvídková J. (2011). Changes of plasma obestatin, ghrelin and NPY in anorexia and bulimia nervosa patients before and after a high-carbohydrate breakfast. Physiol. Res..

[77] Sedlackova D., Kopeckova J., Papezova H., Hainer V., Kvasnickova H., Hill M., Nedvidkova J. (2012). Comparison of a high-carbohydrate and high-protein breakfast effect on plasma ghrelin, obestatin, NPY and PYY levels in women with anorexia and bulimia nervosa. Nutr. Metab..

[78] Galusca B., Prévost G., Germain N., Dubuc I., Ling Y., Anouar Y., Estour B., Chartrel N. (2015). Neuropeptide Y and α-MSH circadian levels in two populations with low body weight: Anorexia nervosa and constitutional thinness. PLoS ONE.

[79] Corder K.M., Li Q., Cortes M.A., Bartley A.F., Davis T.R., Dobrunz L.E. (2020). Overexpression of neuropeptide Y decreases responsiveness to neuropeptide Y. Neuropeptides.

[80] Tam F.I., Seidel M., Boehm I., Ritschel F., Bahnsen K., Biemann R., Weidner K., Roessner V., Ehrlich S. (2020). Peptide YY3–36 concentration in acute- and long-term recovered anorexia nervosa. Eur. J. Nutr..

[81] Rigamonti A.E., Cella S.G., Bonomo S.M., Mancia G., Grassi G., Perotti M., Agosti F., Sartorio A., Muller E.E., Pincelli A.I. (2011). Effect of somatostatin infusion on peptide YY secretion: Studies in the acute and recovery phase of anorexia nervosa and in obesity. Eur. J. Endocrinol..

[82] Stock S., Leichner P., Wong A.C.K., Ghatei M.A., Kieffer T.J., Bloom S.R., Chanoine J.-P. (2005). Ghrelin, peptide YY, glucose-dependent insulinotropic polypeptide, and hunger responses to a mixed meal in anorexic, obese, and control female adolescents. J. Clin. Endocrinol. Metab..

[83] Mancuso C., Izquierdo A., Slattery M., Becker K.R., Plessow F., Thomas J.J., Eddy K.T., Lawson E.A., Misra M. (2020). Changes in appetite-regulating hormones following food intake are associated with changes in reported appetite and a measure of hedonic eating in girls and young women with anorexia nervosa. Psychoneuroendocrinology.

[84] Pfluger P.T., Kampe J., Castaneda T.R., Vahl T., D’Alessio D.A., Kruthaupt T., Benoit S.C., Cuntz U., Rochlitz H.J., Moehlig M. (2006). Effect of Human Body Weight Changes on Circulating Levels of Peptide YY and Peptide YY3–36. J. Clin. Endocrinol. Metab..

[85] Patel B.P., Anderson G.H., Vien S., Bellissimo N., McCrindle B.W., Hamilton J. (2014). Obesity, sex and pubertal status affect appetite hormone responses to a mixed glucose and whey protein drink in adolescents. Clin. Endocrinol..

[86] Bacha F., Arslanian S.A. (2006). Ghrelin and peptide YY in youth: Are there race-related differences?. J. Clin. Endocrinol. Metab..

[87] Mittelman S.D., Klier K., Braun S., Azen C., Geffner M.E., Buchanan T.A. (2010). Obese adolescents show impaired meal responses of the appetite-regulating hormones ghrelin and PYY. Obesity.

[88] Lomenick J.P., Clasey J.L., Anderson J.W. (2008). Meal-related changes in ghrelin, peptide YY, and appetite in normal weight and overweight children. Obesity.

[89] Sysko R., Devlin M.J., Schebendach J., Tanofsky-Kraff M., Zimmerli E., Korner J., Yanovski J.A., Zitsman J.L., Walsh B.T. (2013). Hormonal responses and test meal intake among obese teenagers before and after laparoscopic adjustable gastric banding. Am. J. Clin. Nutr..

[90] Misra M., Tsai P.M., Mendes N., Miller K.K., Klibanski A. (2009). Increased carbohydrate induced ghrelin secretion in obese vs. normal-weight adolescent girls. Obesity.

[91] Roth C., Enriori P.J., Harz K., Woelfle J., Cowley M.A., Reinehr T. (2005). Peptide YY is a regulator of energy homeostasis in obese children before and after weight loss. J. Clin. Endocrinol. Metab..

[92] Gueugnon C., Mougin F., Nguyen N.U., Bouhaddi M., Nicolet-Guénat M., Dumoulin G. (2011). Ghrelin and PYY levels in adolescents with severe obesity: Effects of weight loss induced by long-term exercise training and modified food habits. Eur. J. Appl. Physiol..

[93] Bartolomé M.A., Borque M., Martinez-Sarmiento J., Aparicio E., Hernández C., Cabrerizo L., Fernández-Represa J. (2002). Peptide YY secretion in morbidly obese patients before and after vertical banded gastroplasty. Obes. Surg..

[94] Korner J., Inabnet W., Conwell I.M., Taveras C., Daud A., Olivero-Rivera L., Restuccia N.L., Bessler M. (2006). Differential effects of gastric bypass and banding on circulating gut hormone and leptin levels. Obesity.

[95] Kim B.-J., Carlson O.D., Jang H.-J., Elahi D., Berry C., Egan J.M. (2005). Peptide YY is secreted after oral glucose administration in a gender-specific manner. J. Clin. Endocrinol. Metab..

[96] Holdstock C., Zethelius B., Sundbom M., Karlsson F.A., Engström B.E. (2008). Postprandial changes in gut regulatory peptides in gastric bypass patients. Int. J. Obes..

[97] Roque M.I.V., Camilleri M., Stephens D.A., Jensen M.D., Burton D.D., Baxter K.L., Zinsmeister A.R. (2006). Gastric sensorimotor functions and hormone profile in normal weight, overweight, and obese people. Gastroenterology.

[98] Izquierdo A.G., Crujeiras A.B., Casanueva F.F., Carreira M.C. (2019). Leptin, obesity, and leptin resistance: Where are we 25 years later?. Nutrients.

[99] Karra E., Chandarana K., Batterham R.L. (2009). The role of peptide YY in appetite regulation and obesity. J. Physiol..

[100] Horner K., Lee S. (2015). Appetite-related peptides in childhood and adolescence: Role of ghrelin, PYY, and GLP-1. Appl. Physiol. Nutr. Metab..

[101] Malva J.O., Xapelli S., Baptista S., Valero J., Agasse F., Ferreira R., Silva A. (2012). Multifaces of neuropeptide Y in the brain—Neuroprotection, neurogenesis and neuroinflammation. Neuropeptides.

[102] Miragaia A.S., Wertheimer G.S.D.O., Consoli A.C., Cabbia R., Longo B.M., Girardi C.E.N., Suchecki D. (2018). Maternal deprivation increases anxiety- and depressive-like behaviors in an age-dependent fashion and reduces neuropeptide Y expression in the amygdala and hippocampus of male and female young adult rats. Front. Behav. Neurosci..

[103] Tural U., Iosifescu D.V. (2019). Neuropeptide Y in PTSD, MDD, and chronic stress: A systematic review and meta-analysis. J. Neurosci. Res..

[104] Lach G., De Lima T.C. (2013). Role of NPY Y1 receptor on acquisition, consolidation and extinction on contextual fear conditioning: Dissociation between anxiety, locomotion and non-emotional memory behavior. Neurobiol. Learn. Mem..

[105] Wu G., Feder A., Hagit C., Kim J.J., Calderon S., Charney D., A Mathé A. (2013). Understanding resilience. Front. Behav. Neurosci..

[106] Södersten P., Nergårdh R., Bergh C., Zandian M., Scheurink A. (2008). Behavioral neuroendocrinology and treatment of anorexia nervosa. Front. Neuroendocr..

[107] Baranowska B., Radzikowska M., Wasilewska-Dziubińska E., Kapliński A., Roguski K., Płonowski A. (1999). Neuropeptide Y, leptin, galanin and insulin in women with polycystic ovary syndrome. Gynecol. Endocrinol..

[108] Milewicz A., Mikulski E., Bidzińska B. (2000). Plasma insulin, cholecystokinin, galanin, neuropeptide Y and leptin levels in obese women with and without type 2 diabetes mellitus. Int. J. Obes..

[109] Siva Z.O., Uluduz D., Keskin F.E., Erenler F., Balci H., Uygunoğlu U., Saip S., Göksan B., Siva A., Balci H. (2017). Determinants of glucose metabolism and the role of NPY in the progression of insulin resistance in chronic migraine. Cephalalgia.

[110] Lucka A., Wysokiński A. (2019). Association between adiposity and fasting serum levels of appetite-regulating peptides: Leptin, neuropeptide Y, desacyl ghrelin, peptide YY(1–36), obestatin, cocaine and amphetamine-regulated transcript, and agouti-related protein in nonobese participants. Chin. J. Physiol..

